# Supplementary material for: Reductive evolution and unique predatory mode in the CPR bacterium Vampirococcus lugosii
Source: Nat Commun. 2021 Apr 28;12:2454. doi: 10.1038/s41467-021-22762-4 (PMC8080830; doi:10.1038/s41467-021-22762-4)
Supplement: Supplementary file 1 — Supplementary Information [file 41467_2021_22762_MOESM1_ESM.pdf]

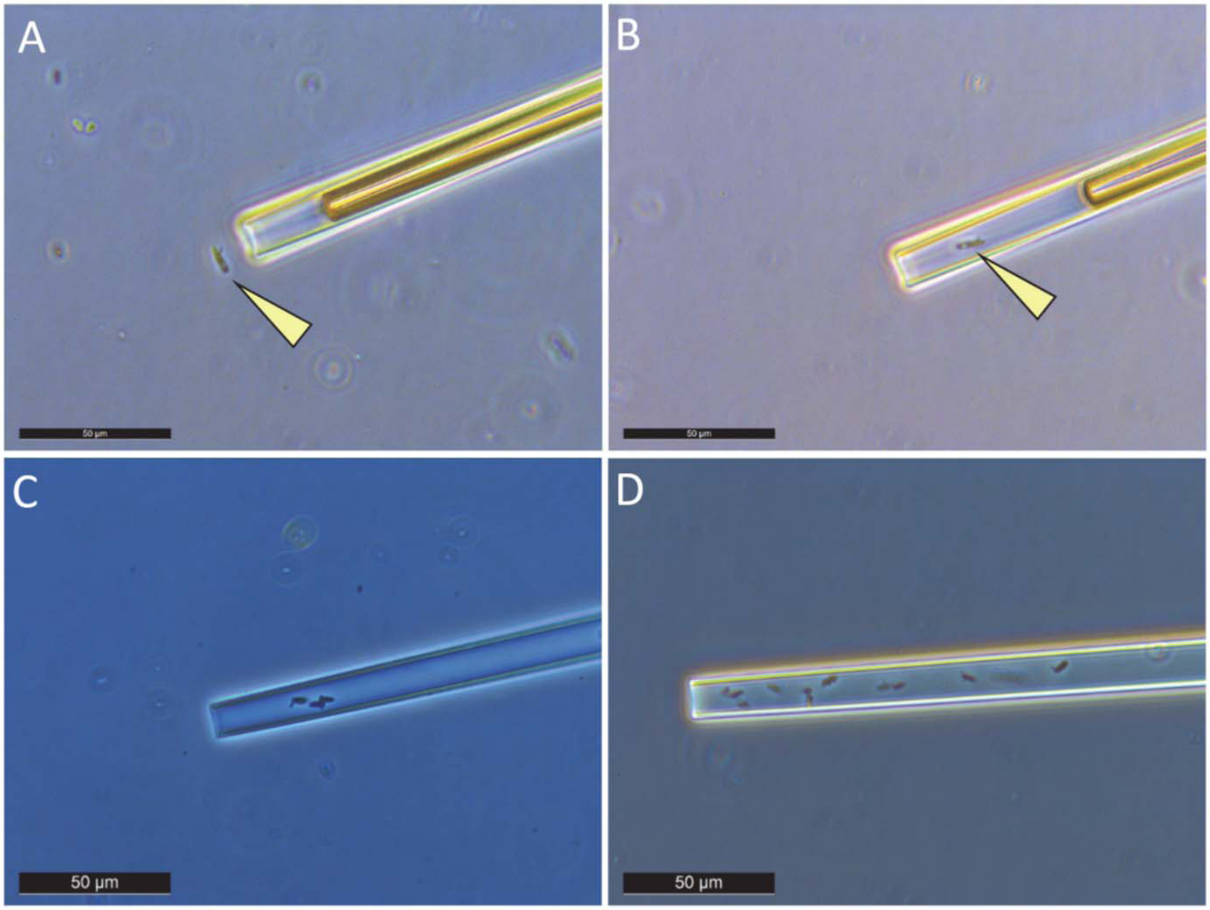

Supplementary Fig. 1| Phase contrast microscopy images of micromanipulation of cells infected by *Vampirococcus* using a glass capillary. Yellow arrows in panels A and B indicate the same infected cells outside and inside the capillary. Panels C and D show capillaries containing several infected cells. We collected 3 sets of 10 *Vampirococcus*+host cells that were used for independent whole genome amplification experiments.

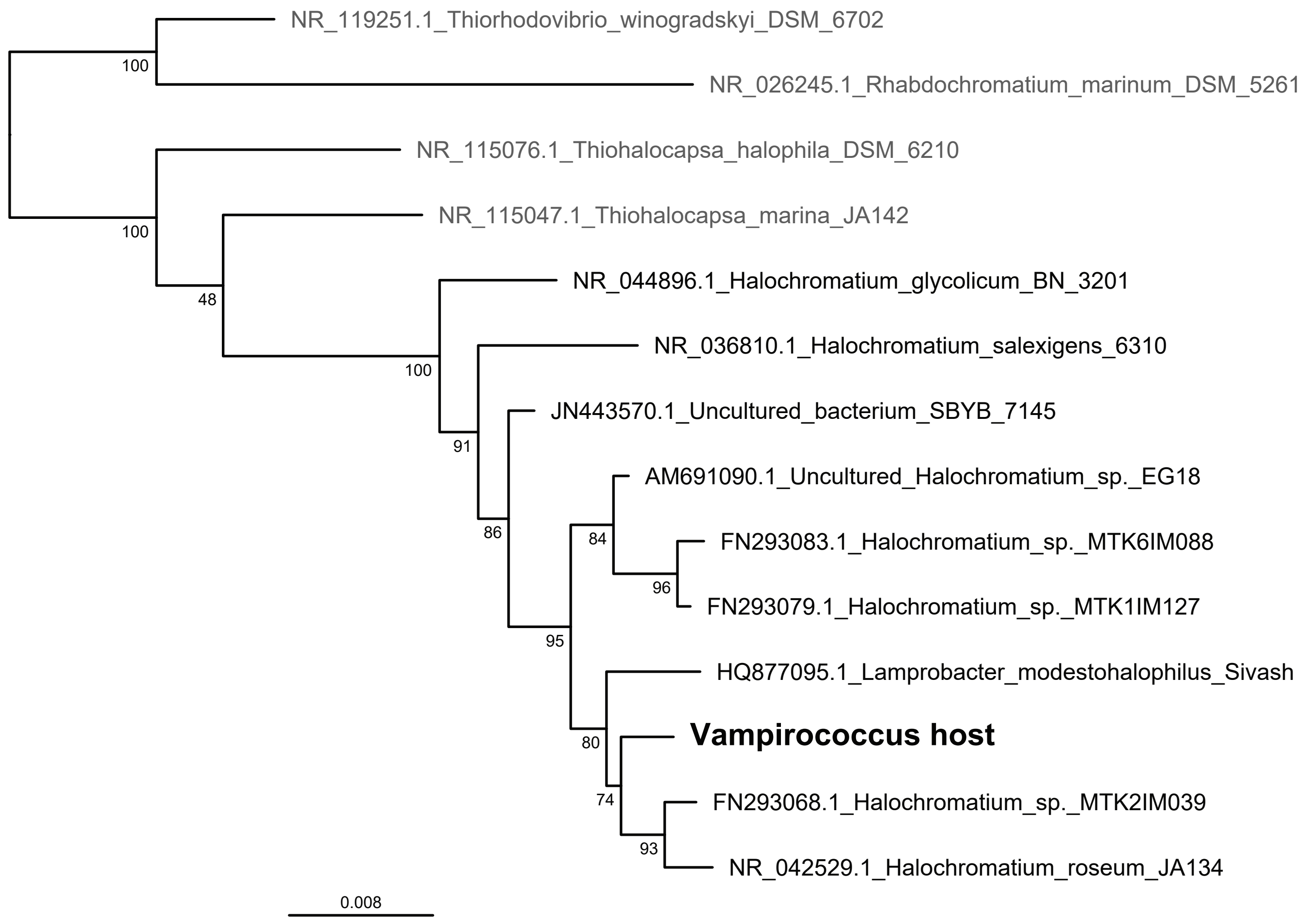

Supplementary Fig. 2| 16S rRNA gene maximum likelihood phylogenetic tree of the *Vampirococcus* host. The tree is based on 1425 conserved aligned positions. Numbers at nodes are bootstrap support values (100 replicates).

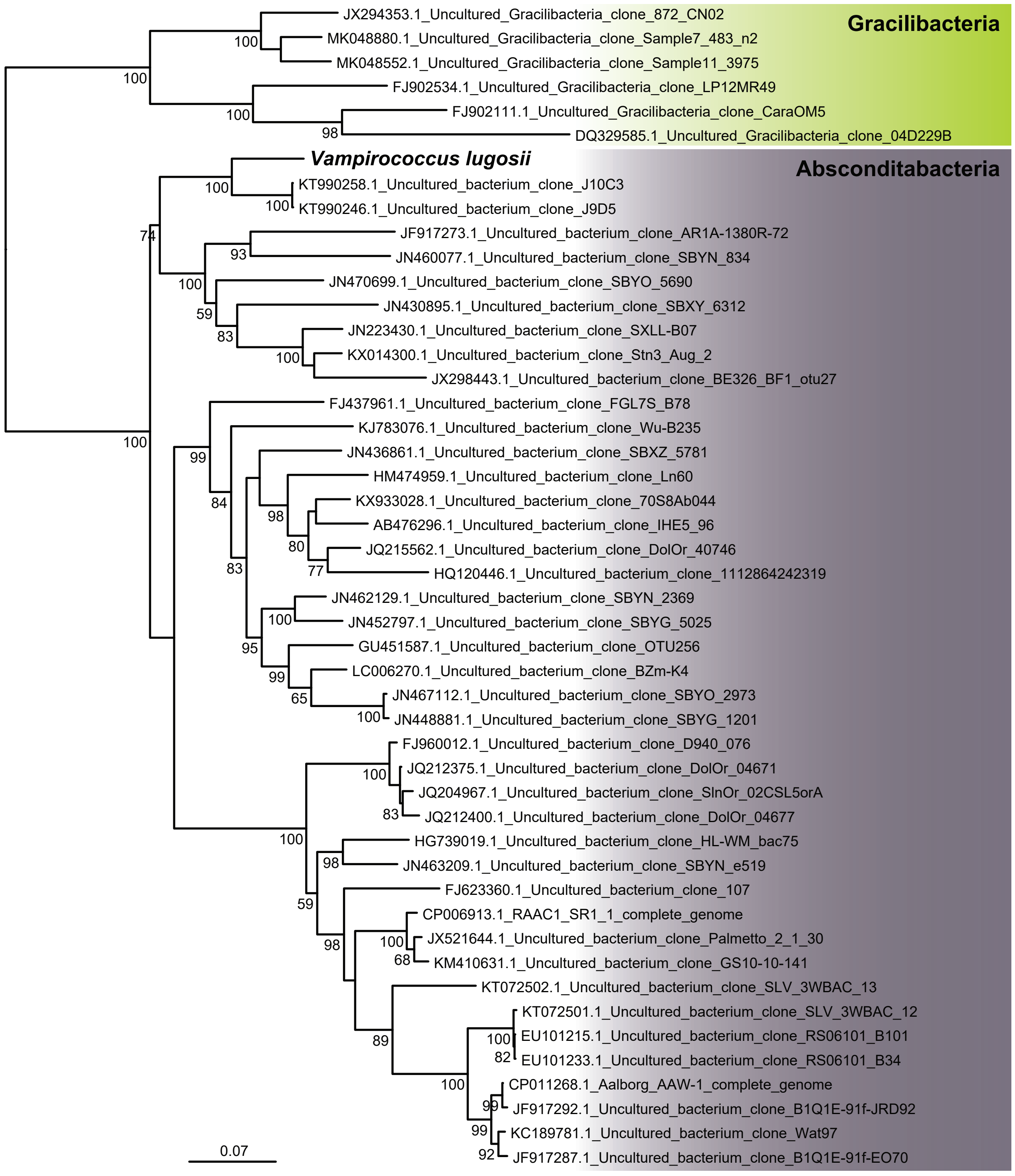

Supplementary Fig. 3| 16S rRNA gene maximum likelihood phylogenetic tree of *Vampirococcus*. The tree is based on 1282 conserved aligned positions. Numbers at nodes are bootstrap support values (100 replicates). The tree is rooted on several Gracilibacteria sequences.

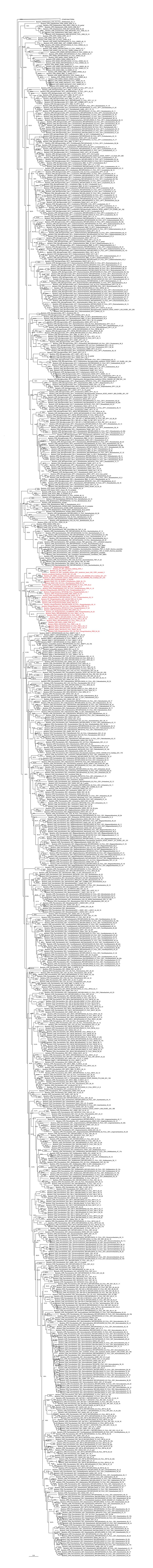

Supplementary Fig. 4| Maximum likelihood phylogenetic tree of CPR bacteria showing the position of *Vampirococcus*. The tree is based on the concatenation of 16 ribosomal proteins and rooted on other bacteria. Numbers at nodes are bootstrap support values (100 replicates). The group containing *Vampirococcus* is indicated in red.

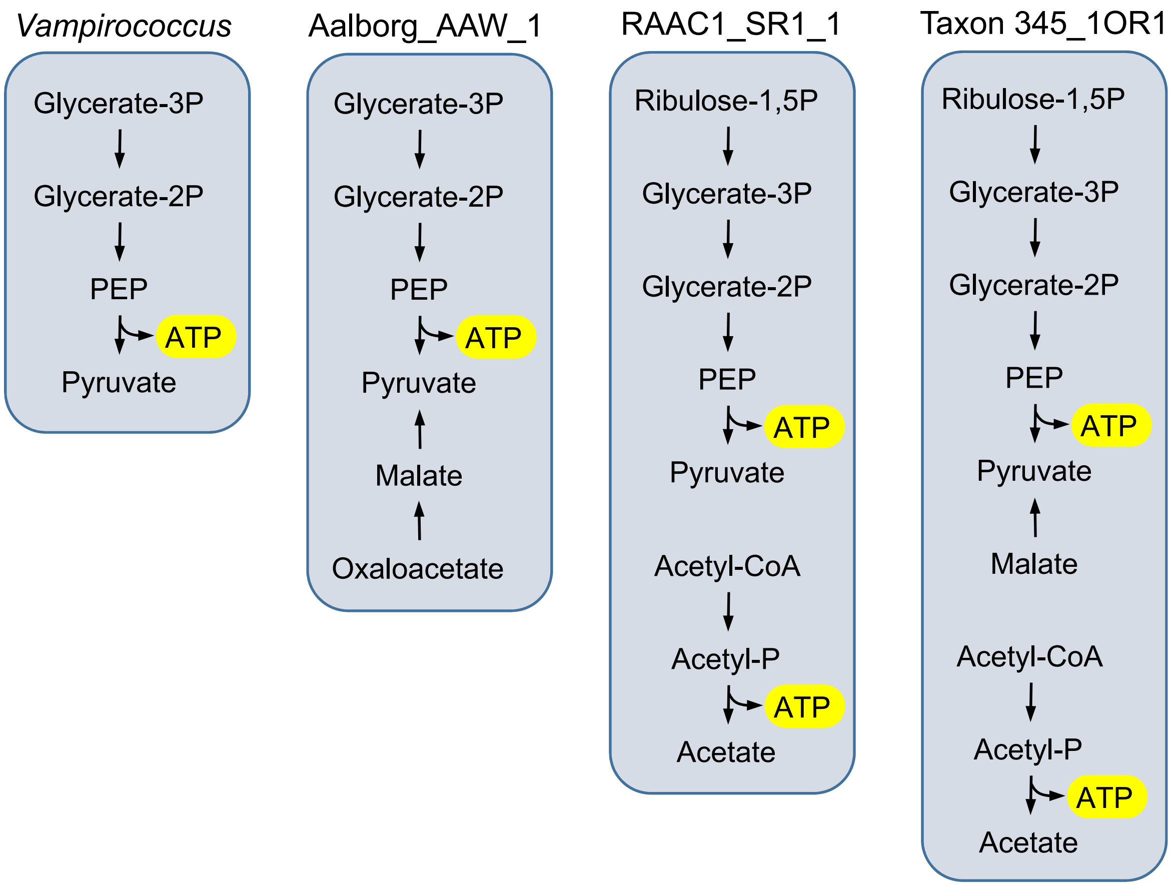

Supplementary Fig. 5| ATP production pathways in *Vampirococcus* and three representative Absconditabacteria.

Acetobacterium woodii	GLAMVAGVGPGIGQGFAAGKGAEAVGKNPTKSNDIVMIMLLGAAVAETSGIFSLVIALILLFANP	(Na^+-dependent)
Ilyobacter tartaricus	GTAMIAGIGPGVGQGYAAGKAVESVARQPEAKGDIISTMVLGQAVAESTGIYSLVIALILLYANP	(Na^+-dependent)
Propionigenium modestum	GAAMIAGIGPGVGQGYAAGKAVESVARQPEAKGDIISTMVLGQAIAESTGIYSLVIALILLYANP	(Na^+-dependent)
Fusobacterium nucleatum	GLAMIAGLGPGIGEGYAAGKAVESVARQPEARGSIISTMILGQAVAESTGIYSLVIALILLYANP	(Na^+-dependent)
Vampirococcus lugosii	LAIWLSWLWVWIWQGRLIVWALWAINRNPEIKNKIMTFMVLFIALIEVTAIYWLIIVFNIMWWLE	?
Thermacetogenium phaeum	LVMGIATIGPALGQGTAAAKALEGMSRQPEMSGELRTTLIIAMAFMEALTIYGLLIAFLLLGKMG	(H^+-dependent)
Synechococcus elongatus	LAVGLAAIGPGIGQGSAAGQAVEGIARQPEAEGKIRGTLLLSLAFMEALTIYGLVVALVLLFANP	(H^+-dependent)
Vibrio cholera	IIVGLCAVGTAIGFAVLGGKFLEGAARQPEMAPMLQVKMFIIAGLLDAVPMIGIVIALLFTFANP	(H^+-dependent)
Escherichia coli	VMMGLAAIGAAIGIGILGGKFLEGAARQPDLIPLLRTQFFIVMGLVDAIPMIAVGLGLYVMFAVA	(H^+-dependent)
Bacillus subtilis	IAIGLGALGAGIGNGLIVSRTVEGIARQPEAGKELRTLMFMGIALVEALPIIAVVIAFLAFFG..	(H^+-dependent)

Supplementary Fig. 6 | Alignment of the region of the subunit c of the ATP synthase containing the Na+-binding motif in the Na+-dependent enzymes. The amino-acids of the Na+-binding motif are highlighted in color (those that have amino acids shared with the H+-dependent enzymes in green and those that have different amino acids in orange). Notice that *Vampirococcus* has two positions with differences with the Na+-dependent enzymes, suggesting that it has a H+-dependent ATP synthase.

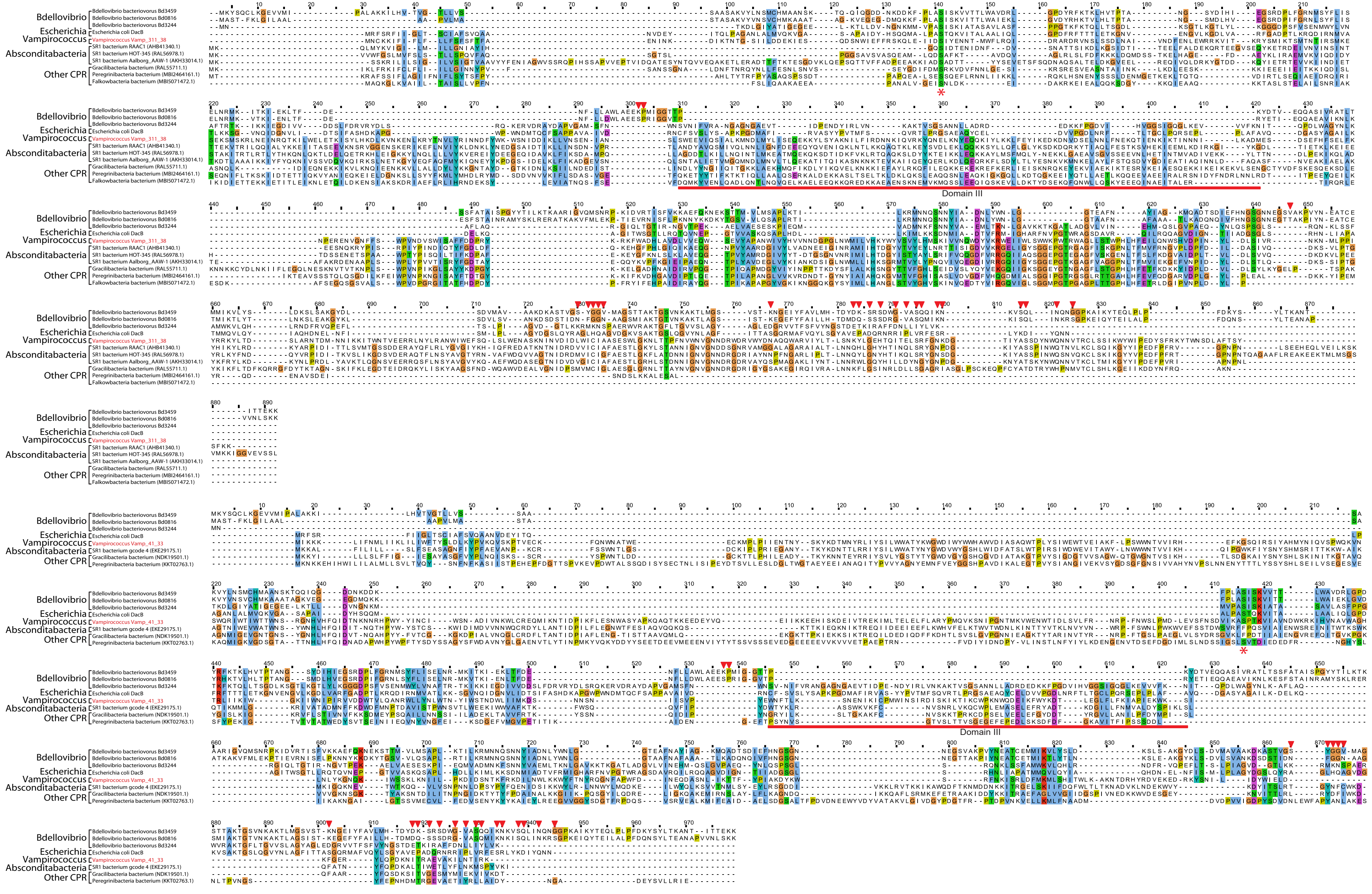

Supplementary Fig. 7| Alignment of the *Vampirococcus* DD endopeptidases with those of other bacteria, including the *Bdellovibrio* 'predatory' (Bd3459 and Bd0816) and self-wall (Bd3244) endopeptidases. The acive site serine residue in indicated with a red asterisk, the regulatory domain III is underlined in red, and the positions that interact in the *Bdellovibrio* 'predatory' endopeptidases with the self-protective inhibitor Bd3460 are marked with red triangles. Amino acids are coloured according to the 'clustal' color palette implemented in Jalview.

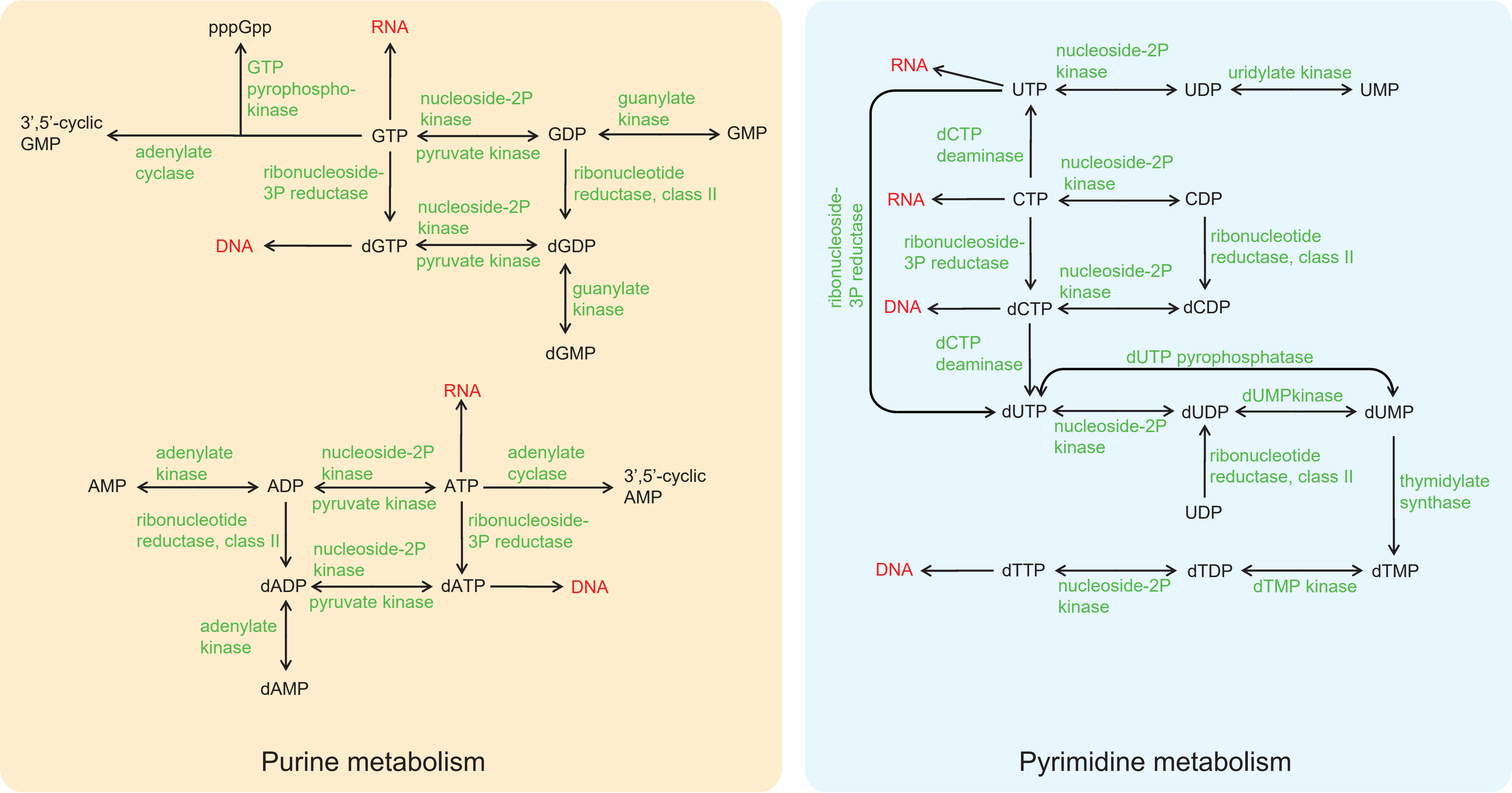

Supplementary Fig. 8| Main pathways involved in purine and pyrimidine metabolism in *Vampirococcus*. Substrates are indicated in black, enzymes in green, and DNA and RNA in red.

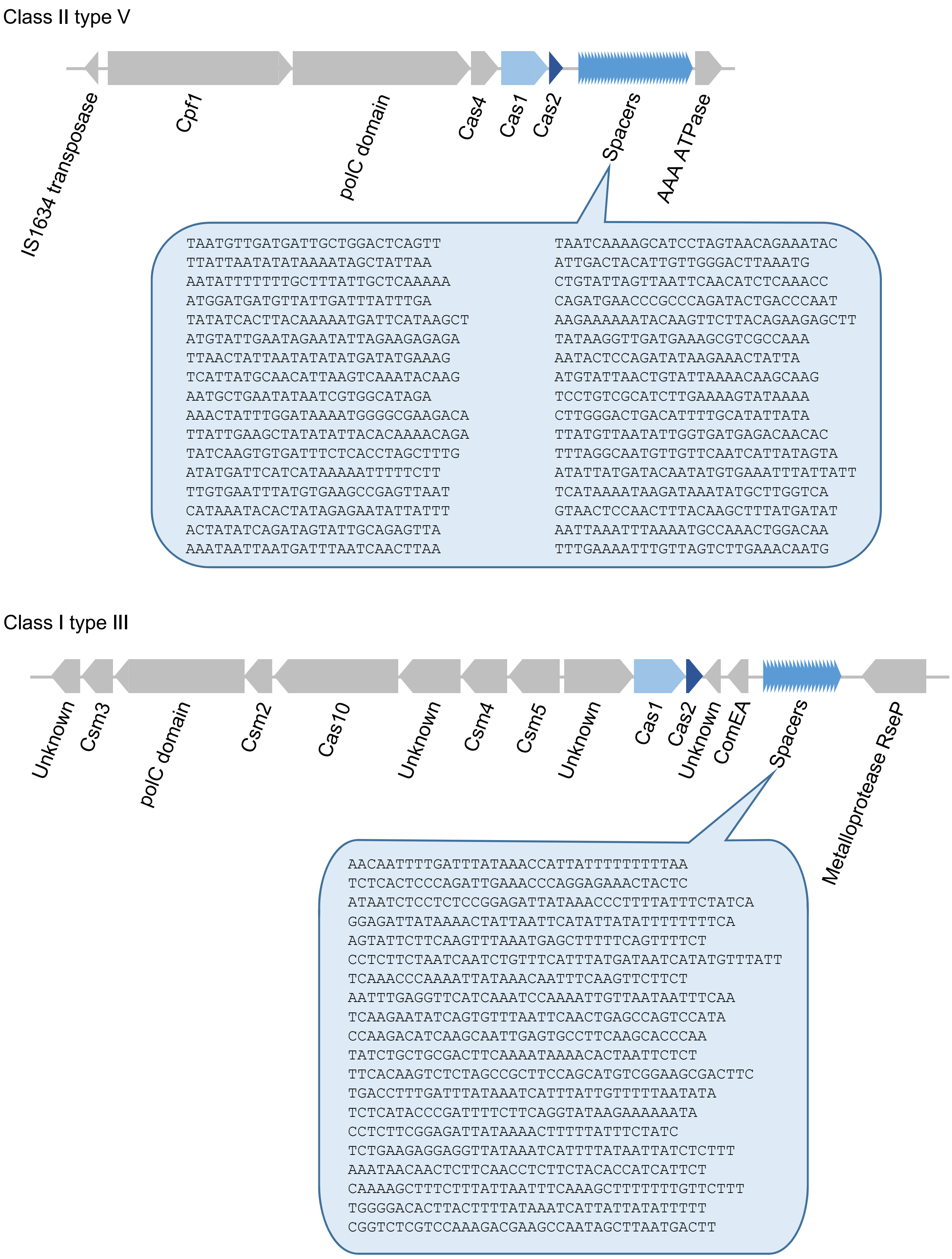

Supplementary Fig. 9| The two CRISPR-Cas loci of *Vampirococcus* and their corresponding spacer sequences. Elements that are shared by the two loci are indicated in blue.

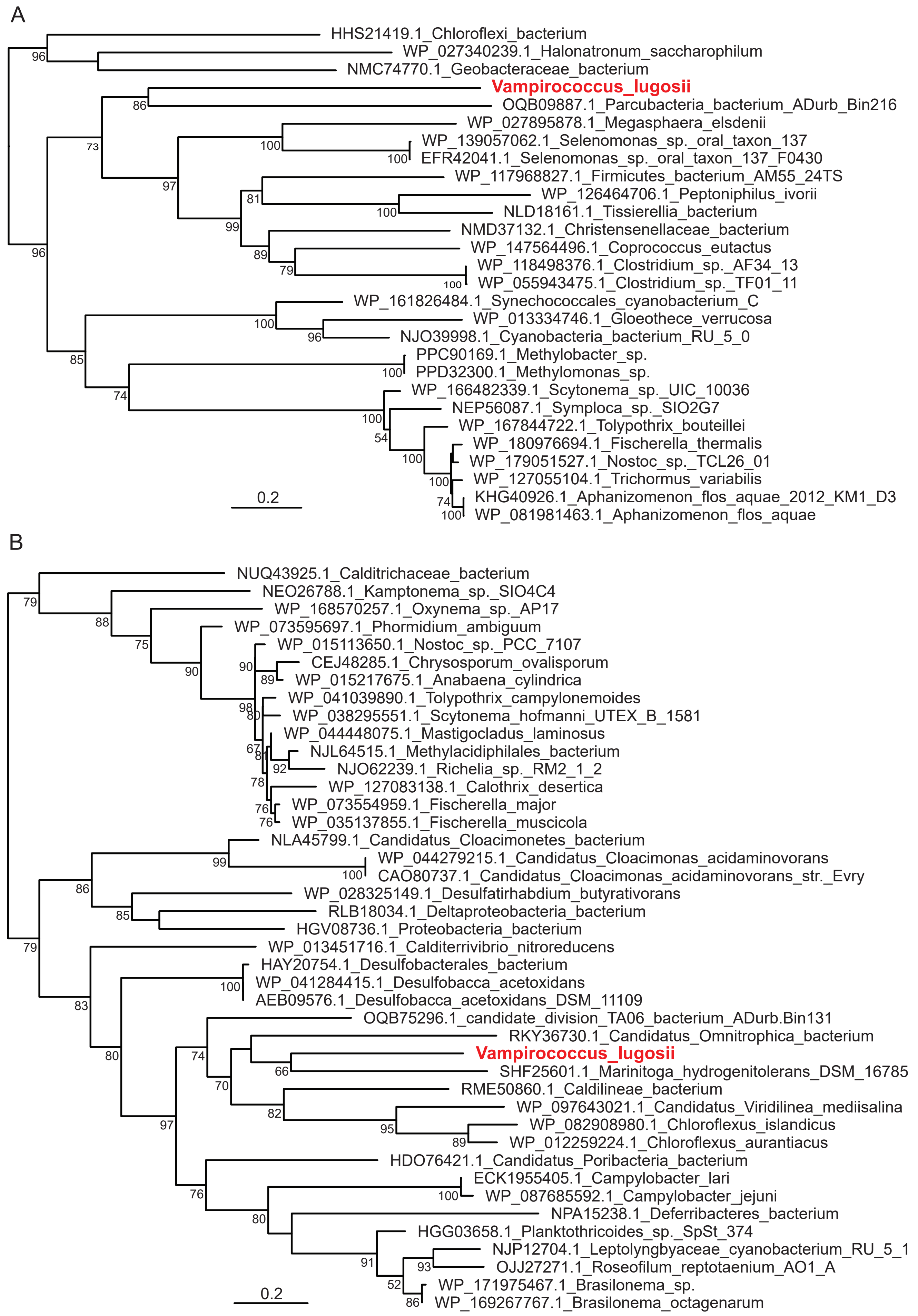

Supplementary Fig. 10| Maximum likelihood phylogenetic trees of *Vampirococcus* type I class III CRISPR-Cas enzymes Cas1 ‘A) and Cas2 (B). The trees are based on 349 and 118 conserved aligned positions, respectively. Numbers at nodes are bootstrap support values (100 replicates, only values >50% are shown).

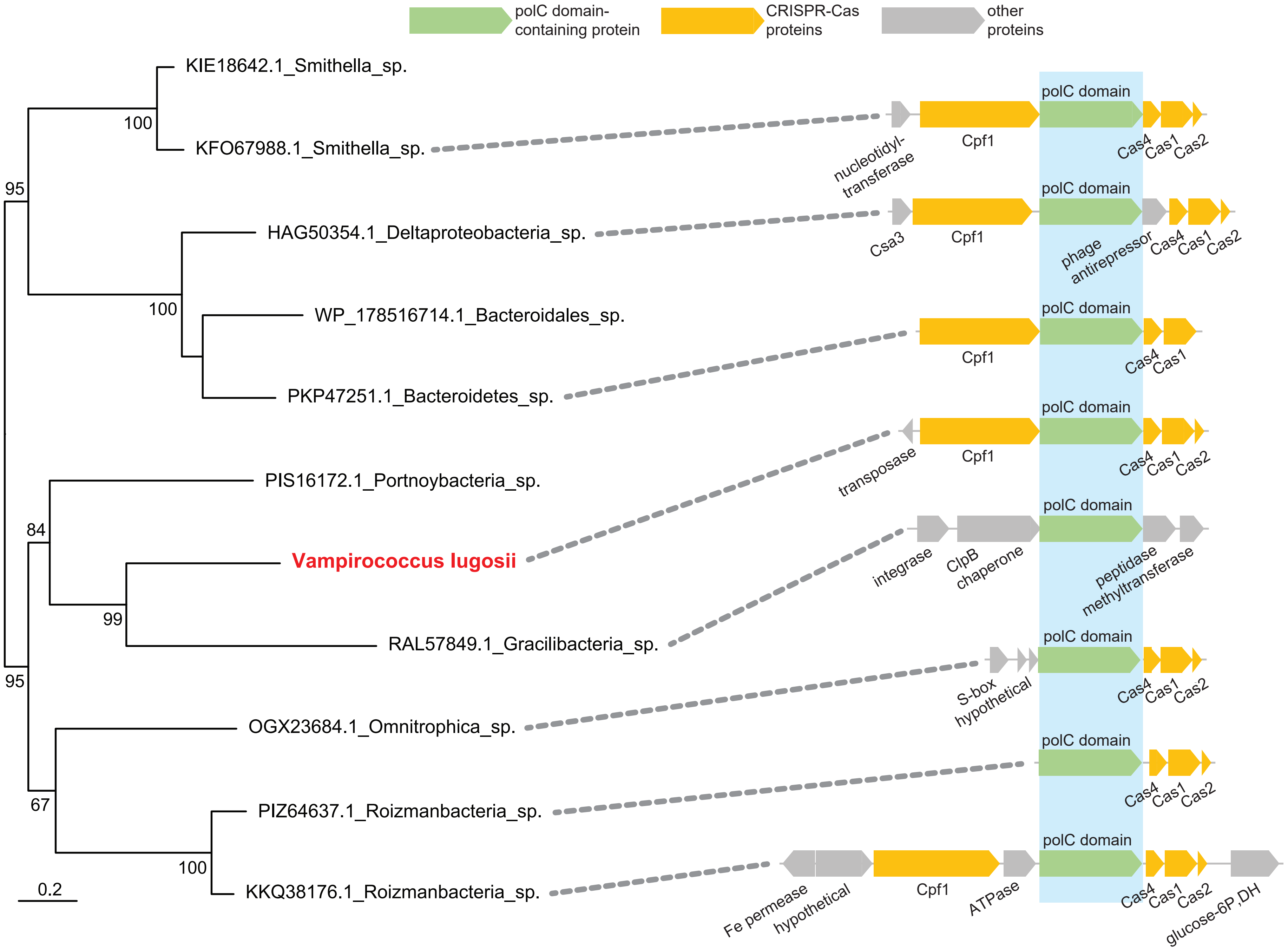

Supplementary Fig. 11| Maximum likelihood phylogeny and genomic context of the *Vampirococcus* polC domain-containing protein associated to the class II type V CRISPR-Cas system. The genomic context is shown for the species for which genome sequence data are available. The polC domain-containing protein is shown in green and typical CRISPR-Cas proteins are shown in orange.

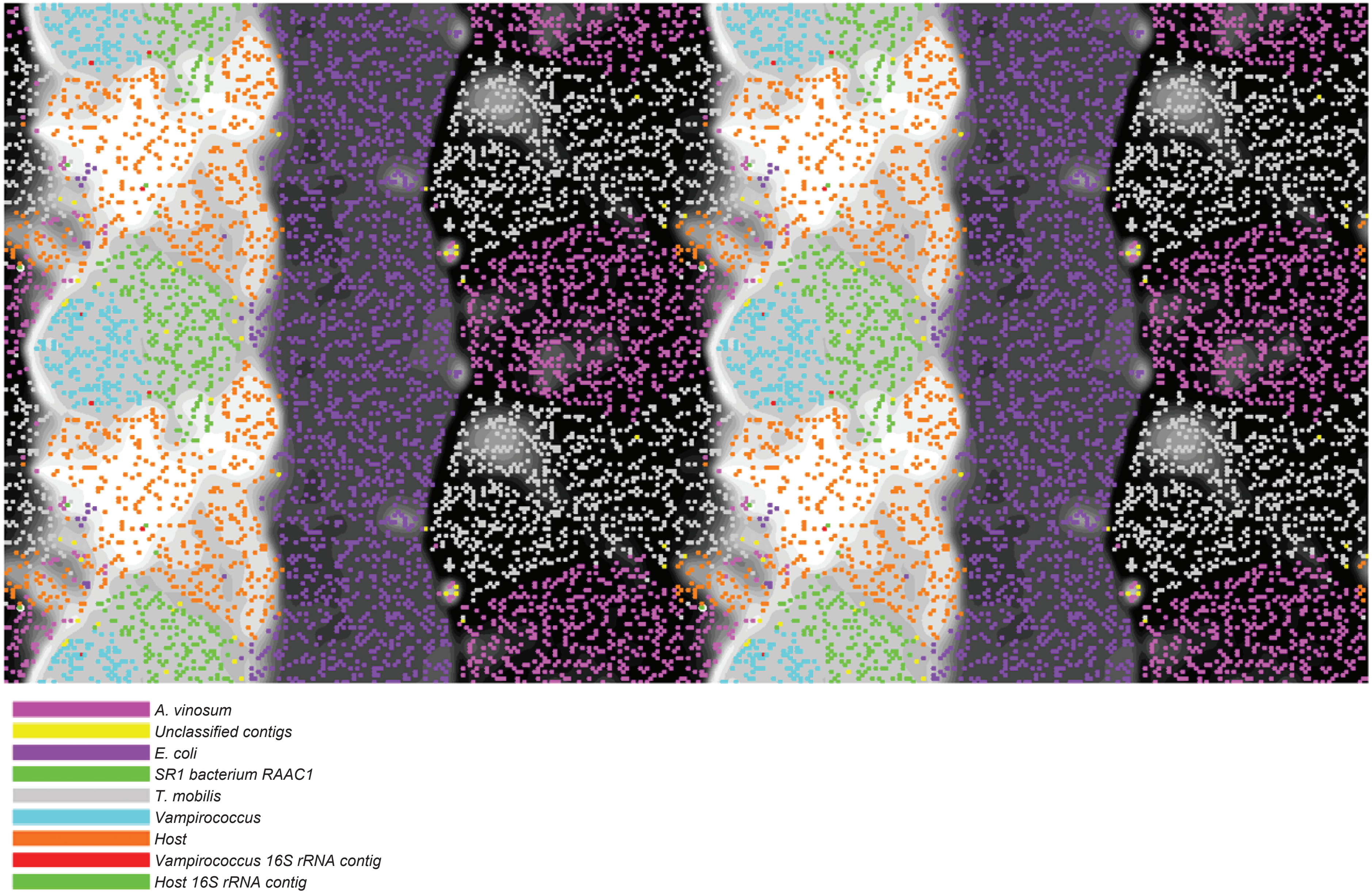

Supplementary Fig. 12 | Visualization of the ESOM map of the assembled DNA contigs of *Vampirococcus*, its host, and several reference genomes.

Protein	Function	Size
VAMP_16_20	hypothetical protein	4163
VAMP_134_102	NAD(P)-dependent dehydrogenase	2400
VAMP_6_203	alpha-2 macroglobulin	2368
VAMP_166_2	hypothetical protein	2284
VAMP_19_245	alpha-2 macroglobulin	1895
VAMP_328_89	membrane protein	1883
VAMP_95_65	YfaS, alpha-2 macroglobulin family	1871
VAMP_54_69	hypothetical protein	1723
VAMP_17-88-45_126	PKD repeat-containing protein	1396
VAMP_98_96	DNA-binding beta propeller protein YncE	1392

Supplementary Table 1| Ten largest proteins encoded in the ***Vampirococcus*** **genome.**

Primer name	Target	Sequence
B-27F	16S rRNA gene	AGAGTTTGATCCTGGCTCAG
1492R	16S rRNA gene	GGTTACCTTGTTACGACTT

Supplementary Table 2| Primers used in our study.
