# Supplementary material for: Reductive evolution and unique predatory mode in the CPR bacterium Vampirococcus lugosii
Source: Nat Commun. 2021 Apr 28;12:2454. doi: 10.1038/s41467-021-22762-4 (PMC8080830; doi:10.1038/s41467-021-22762-4)
Supplement: Supplementary file 2 — Description of Additional Supplementary Files [file 41467_2021_22762_MOESM2_ESM.pdf]

Description of Additional Supplementary Files

File Name: Supplementary Movie 1
Description: This movie shows several *Halochromatium* sp. cells infected by *Vampirococcus*. *Halochromatium* cells are bigger, flagellated, and contain conspicuous refringent sulfur granules in their cytoplasm. *Vampirococcus* cells are the small, darker cells attached to the surface of *Halochromatium* cells.

File Name: Supplementary Data 1
Description: *Vampirococcus* genes acquired by horizontal gene transfer.
