# Supplementary material for: Reductive evolution and unique predatory mode in the CPR bacterium Vampirococcus lugosii
Source: Nat Commun. 2021 Apr 28;12:2454. doi: 10.1038/s41467-021-22762-4 (PMC8080830; doi:10.1038/s41467-021-22762-4)
Supplement: Supplementary file 5 — Reporting Summary [file 41467_2021_22762_MOESM5_ESM.pdf]

nature research

Corresponding author(s): David Moreira

Last updated by author(s): Mar 3, 2021

# Reporting Summary

Nature Research wishes to improve the reproducibility of the work that we publish. This form provides structure for consistency and transparency in reporting. For further information on Nature Research policies, see our Editorial Policies and the Editorial Policy Checklist.

## Statistics

For all statistical analyses, confirm that the following items are present in the figure legend, table legend, main text, or Methods section.

| n/a | Confirmed | |
|---|---|---|
| ☒ | ☐ | The exact sample size (*n*) for each experimental group/condition, given as a discrete number and unit of measurement |
| ☒ | ☐ | A statement on whether measurements were taken from distinct samples or whether the same sample was measured repeatedly |
| ☒ | ☐ | The statistical test(s) used AND whether they are one- or two-sided<br>*Only common tests should be described solely by name; describe more complex techniques in the Methods section.* |
| ☒ | ☐ | A description of all covariates tested |
| ☒ | ☐ | A description of any assumptions or corrections, such as tests of normality and adjustment for multiple comparisons |
| ☒ | ☐ | A full description of the statistical parameters including central tendency (e.g. means) or other basic estimates (e.g. regression coefficient) AND variation (e.g. standard deviation) or associated estimates of uncertainty (e.g. confidence intervals) |
| ☒ | ☐ | For null hypothesis testing, the test statistic (e.g. *F*, *t*, *r*) with confidence intervals, effect sizes, degrees of freedom and *P* value noted<br>*Give P values as exact values whenever suitable.* |
| ☒ | ☐ | For Bayesian analysis, information on the choice of priors and Markov chain Monte Carlo settings |
| ☒ | ☐ | For hierarchical and complex designs, identification of the appropriate level for tests and full reporting of outcomes |
| ☒ | ☐ | Estimates of effect sizes (e.g. Cohen's *d*, Pearson's *r*), indicating how they were calculated |

*Our web collection on statistics for biologists contains articles on many of the points above.*

## Software and code

Policy information about availability of computer code

| | |
|---|---|
| Data collection | DNA and protein sequences similar to those of Vampirococcus were identified and downloaded from the GenBank public database using the NCBI online blastn and blastp tools, respectively. |
| Data analysis | Sequence assembly: SPAdes v3.6.0<br>Contig clustering and ESOM map verification: ESOM Tools v1.1<br>Nucleotide tetramer frequency distributions calculation: tetramer_freqs_esom.pl script<br>Genome completeness and contamination estimation: CheckM v1.0.18<br>CDS prediction: Prodigal v2.6.2<br>Sequence similarity searches: DIAMOND v0.7.9<br>Conserved protein motifs search: SMART v8.0<br>Prediction of transmembrane helices: TMHMM Server v2.0<br>Detection of CRISPR-Cas loci: CRISPRCasFinder v1 and CRISPRminer v1<br>Detection of orthologous genes: OrthoFinder v1.1.20<br>Gene gain/loss calculation: Count v10.04<br>Sequence alignment: MAFFT V7<br>Alignment trimming: trimAl v1.2<br>Phylogenetic tree reconstruction: IQ-tree v1.5.549<br>Concatenation of protein alignments: SequenceMatrix v1.8 |

For manuscripts utilizing custom algorithms or software that are central to the research but not yet described in published literature, software must be made available to editors and reviewers. We strongly encourage code deposition in a community repository (e.g. GitHub). See the Nature Research guidelines for submitting code & software for further information.

## Data

Policy information about availability of data

All manuscripts must include a data availability statement. This statement should provide the following information, where applicable:

- Accession codes, unique identifiers, or web links for publicly available datasets
- A list of figures that have associated raw data
- A description of any restrictions on data availability

Sequence data have been deposited in GenBank with the following accession numbers: MW286273 [https://www.ncbi.nlm.nih.gov/nuccore/MW286273] and MW286274 [https://www.ncbi.nlm.nih.gov/nuccore/MW286274] (Vampirococcus and Halochromatium host 16S rRNA gene sequences, respectively) and PRJNA678638 [https://www.ncbi.nlm.nih.gov/bioproject/?term=PRJNA678638] (Vampirococcus genome sequence). We used the following databases for our analyses: KEGG (release 93.0; Jan 1, 2020; https://www.genome.jp/kegg/), RefSeq (release 68; Nov 3, 2014; https://www.ncbi.nlm.nih.gov/refseq/), COG (release 2014; Dec 2014; https://www.ncbi.nlm.nih.gov/research/cog-project/), and SEED (release Sept 14, 2011; https://theseed.org/wiki/DownloadPage).

# Field-specific reporting

Please select the one below that is the best fit for your research. If you are not sure, read the appropriate sections before making your selection.

☐ Life sciences ☐ Behavioural & social sciences ☒ Ecological, evolutionary & environmental sciences

For a reference copy of the document with all sections, see nature.com/documents/nr-reporting-summary-flat.pdf

# Ecological, evolutionary & environmental sciences study design

All studies must disclose on these points even when the disclosure is negative.

| | |
|---|---|
| Study description | In this study, we determine the genome sequence of single-picked consortia of the predatory bacterium Vampirococcus lugosii and its anoxygenic  photosyntetic host. Optical and electron microscopy analysis of live samples allow to describe a peculiar mode of infection with multicellular stalks; and nutrition by absorption of released host metabolites. Phylogenomic analysis indicates that V. lugosii is the first identified representative of the Absconditabacteria, one phylum of the Candidate Phyla Radiation (CPR). Comparative genomics with other CPR bacteria show that genome reduction and adaptation to a parasitic lifestyle are shared by Vampirococcus and several related CPR groups. Vampirococcus also possesses many uncharacterized genes and novel CRISPR-Cas systems. |
| Research sample | Microbial mat from athalassic hypersaline lake - Salada de Chiprana - 55.9 g total dissolved salt l–1, pH 8.23. The Salada de Chiprana lake bottom is covered by a thick (~5 cm) and homogeneous microbial mat, we sampled an accessible site close to the shore which was representative of this extensive microbial mat. The size of the sample 20 x 20 x 8 cm allowed to recover all the mat layers and, therefore, the various microbial communities forming them. |
| Sampling strategy | A microbial mat sample was collected by D.M., P.L-G., A.I.L.-A. and M.I. Its large size (20 x 20 x 8 cm) was chosen to assure that all major microbial groups inhabiting the Salada de Chiprana microbial mat were represented. The sample was transported to the laboratory and after three weeks of incubation at room temperature, consortia of Chromatium-like hosts and epibiotic bacteria were observed. Individual consortia were micromanipulated with an Eppendorf PatchMan NP2 micromanipulator equipped with 6 μm-diameter microcapillaries (Eppendorf) mounted on a Leica DIII3000 B inverted microscope. |
| Data collection | Individual consortia were micromanipulated by D.M. with an Eppendorf PatchMan NP2 micromanipulator equipped with 6 μm-diameter microcapillaries (Eppendorf) mounted on a Leica DIII3000 B inverted microscope. Their genomes were MDA-amplified (by D.M. and P.L.-G.) and then sequenced using Illumina HiSeq2500 v4 (Eurofins Genomics; Ebersberg, Germany). |
| Timing and spatial scale | Samples were collected from a single sample point in the Salada de Chiprana in December 14th-15th 2013. Winter was chosen because the lake has high water level and good microbial mat development. The Vampirococcus-host consortia were micromanipulated as soon as they apperaered in our sample (January 2014). Genomes were amplified and sequenced inmediately afterwards (February 2014). |
| Data exclusions | No data were excluded from the analyses. |
| Reproducibility | Three sets of ~10 Vampirococcus+host consortia were used for genome amplification and sequencing usinf MDA (2 sets) and MALBAC (1 set) procedures. All amplification experiments yielded enough DNA for Illumina sequencing. Sequence data from the three independent whole-genome amplifications were obtained and used for genome analysis. |
| Randomization | We did not carry out any statistical analysis that required randomization. |
| Blinding | We did not carry out any analysis that required blinding. |

Did the study involve field work? ☒ Yes ☐ No

## Field work, collection and transport

| | |
|---|---|
| Field conditions | Microbial mats were sampled in December 2013 in the Salada de Chiprana (NE Spain) permanent athalassic hypersaline lake (55.9 g |

| | |
|---|---|
| | total dissolved salt l–1, pH 8.23) at a depth of ~50 cm. The average temperature in that month ranged between 4.5°C (min.) and 15°C (max.) and the cumulated rainfall was of 22.9 l/m2. |
| Location | The Salada de Chiprana lake is located in NE Spain '41°14′22″N 0°11′11″W) at 178 m above sea level. We collected samples on the North shore of the lake, at a depth of ~50 cm. |
| Access & import/export | D.M., P.L-G., A.I.L.-A., and M.I. were granted the permission to collect microbial mat samples in the Salada de Chiprana lake by the "Gobierno de Aragon" (the Regional Government of Aragon, the Spanish region where the lake is located). The permit (number 134944) was issued on July 16th 2013 and was valid until December 31th 2013. We collected our samples in December 14th-15th 2013, within the time framework of the permit. |
| Disturbance | The collected microbial mat fragment had a size of ~20 x 20 x 8 cm. This type of sampling is practically negligible in the general context of the Salada de Chiprana ecosystem, which has a total surface of ~310000 m2. |

# Reporting for specific materials, systems and methods

We require information from authors about some types of materials, experimental systems and methods used in many studies. Here, indicate whether each material, system or method listed is relevant to your study. If you are not sure if a list item applies to your research, read the appropriate section before selecting a response.

## Materials & experimental systems

| n/a | Involved in the study | |
|---|---|---|
| ☒ | ☐ | Antibodies |
| ☒ | ☐ | Eukaryotic cell lines |
| ☒ | ☐ | Palaeontology and archaeology |
| ☒ | ☐ | Animals and other organisms |
| ☒ | ☐ | Human research participants |
| ☒ | ☐ | Clinical data |
| ☒ | ☐ | Dual use research of concern |

## Methods

| n/a | Involved in the study | |
|---|---|---|
| ☒ | ☐ | ChIP-seq |
| ☒ | ☐ | Flow cytometry |
| ☒ | ☐ | MRI-based neuroimaging |
